# Proliferation of East Antarctic Adélie penguins in response to historical deglaciation

**DOI:** 10.1186/s12862-015-0502-2

**Published:** 2015-11-18

**Authors:** Jane Younger, Louise Emmerson, Colin Southwell, Patrick Lelliott, Karen Miller

**Affiliations:** Institute for Marine and Antarctic Studies, University of Tasmania, Private Bag 129, Hobart, 7001 TAS Australia; Australian School of Advanced Medicine, Macquarie University, 2 Technology Place, 2109 NSW Sydney, Australia; Australian Antarctic Division, 203 Channel Highway, Kingston, 7050 TAS Australia; John Curtin School of Medical Research, Australian National University, 131 Garran Road, Acton, 2601 ACT Australia; Australian Institute of Marine Science, The UWA Oceans Institute, The University of Western Australia, 35 Stirling Highway, Crawley, WA 6009 Australia; School of Biological Sciences, Private Bag 5, University of Tasmania, Hobart, 7001 TAS Australia

**Keywords:** Climate change ecology, Bayesian skyline plot, Palaeoecology, Holocene, Molecular ecology, Seabirds, *Pygoscelis adeliae*, Last glacial maximum, Demography

## Abstract

**Background:**

Major, long-term environmental changes are projected in the Southern Ocean and these are likely to have impacts for marine predators such as the Adélie penguin (*Pygoscelis adeliae*). Decadal monitoring studies have provided insight into the short-term environmental sensitivities of Adélie penguin populations, particularly to sea ice changes. However, given the long-term nature of projected climate change, it is also prudent to consider the responses of populations to environmental change over longer time scales. We investigated the population trajectory of Adélie penguins during the last glacial-interglacial transition to determine how the species was affected by climate warming over millennia. We focussed our study on East Antarctica, which is home to 30 % of the global population of Adélie penguins.

**Methods:**

Using mitochondrial DNA from extant colonies, we reconstructed the population trend of Adélie penguins in East Antarctica over the past 22,000 years using an extended Bayesian skyline plot method. To determine the relationship of East Antarctic Adélie penguins with populations elsewhere in Antarctica we constructed a phylogeny using mitochondrial DNA sequences.

**Results:**

We found that the Adélie penguin population expanded 135-fold from approximately 14,000 years ago. The population growth was coincident with deglaciation in East Antarctica and, therefore, an increase in ice-free ground suitable for Adélie penguin nesting. Our phylogenetic analysis indicated that East Antarctic Adélie penguins share a common ancestor with Adélie penguins from the Antarctic Peninsula and Scotia Arc, with an estimated age of 29,000 years ago, in the midst of the last glacial period. This finding suggests that extant colonies in East Antarctica, the Scotia Arc and the Antarctic Peninsula were founded from a single glacial refuge.

**Conclusions:**

While changes in sea ice conditions are a critical driver of Adélie penguin population success over decadal and yearly timescales, deglaciation appears to have been the key driver of population change over millennia. This suggests that environmental drivers of population trends over thousands of years may differ to drivers over years or decades, highlighting the need to consider millennial-scale trends alongside contemporary data for the forecasting of species’ abundance and distribution changes under future climate change scenarios.

## Background

While climate change is a global phenomenon, its environmental effects can vary dramatically in different locations. For example, trends in the extent and duration of sea ice around the Antarctic continent show high spatial heterogeneity [1, 2]. Over a 34 year monitoring period, sea ice extent decreased in the Bellingshausen and Amundsen Seas accompanied by a dramatic shortening of the sea ice season by 100 ± 31 days [1, 3]. Meanwhile, in the Ross Sea, both extent and duration of the sea ice season increased substantially over the same period [1]. Even within Antarctic regions there have been variations in the extent and duration of sea ice. For example, the East Antarctic region, defined here as between 30 and 150°E, has demonstrated considerably more complex trends in sea ice seasonality and extent than the rest of the continent [4, 5]. Since 1980, in some East Antarctic areas (between 95 and 110°E; and isolated pockets between 75 and 150°E), there has been a significant shortening of the sea ice season by up to 93 days [4]. Meanwhile, many other localities (west of 105°E; between 40 and 90°E) have experienced a significant lengthening of the sea ice season [4]. Antarctic sea ice is expected to undergo further declines in the future; in the most extreme climate model scenario (RCP8.5), East Antarctica would be completely free of sea ice in February by 2081–2100, while only small portions of the Weddell and Ross Seas would retain sea ice [6].

**Fig. 1:**
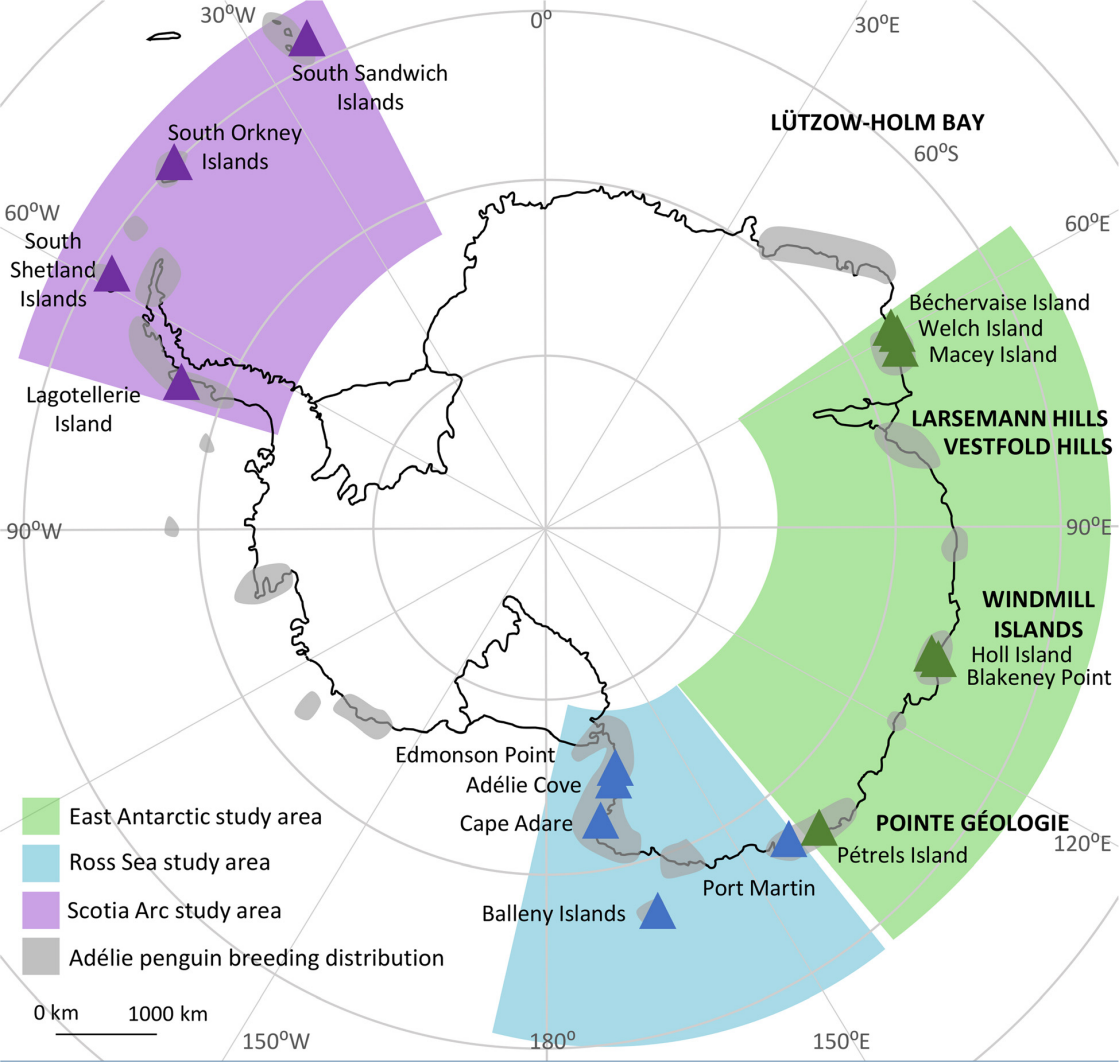
Adélie penguin breeding distribution. Grey shading indicates the approximate extant breeding distribution of Adélie penguins [11, 58]. Green triangles indicate East Antarctic colonies sampled in this study, blue and purple triangles indicate colonies in the Ross Sea [31] and Scotia Arc [29] datasets, respectively

Environmental changes that are currently underway in the Southern Ocean, including changes in the timing of sea ice advance and retreat, receding glaciers, and shifting oceanographic fronts, could lead to major changes in the terrestrial breeding habitats, marine foraging environment and prey availability for higher order predators [7, 8]. The Adélie penguin (*Pygoscelis adeliae*) is a prime example of a Southern Ocean predator that is likely to be affected by environmental impacts associated with the Anthropocene [9]. The species forms breeding colonies on ice-free land along the Antarctic coastline (Fig. 1) [10, 11] and forages largely in the pack ice zone during the breeding season [12]. Adélie penguin populations are known to be sensitive to changes in sea ice extent, the timing of sea ice retreat [13, 14] and the extent of glaciation [15]. Given the large regional variability in the physical manifestations of climate change, it is likely that geographically distant populations of Adélie penguins will experience very different environmental impacts [16].

East Antarctica is home to approximately 30 % of the global population of Adélie penguins, with an estimated abundance of 1.14 million breeding pairs [11]. The breeding distribution in this region has expanded over the past several decades, possibly as a result of sustained population growth [17]. Sea ice conditions strongly influence Adélie penguin populations in East Antarctica, although the mechanisms of impact are complex and depend on the nature, extent and timing of the presence of sea ice [13, 18]. The Béchervaise Island population experienced near total reproductive failure in years with extensive near-shore sea ice during the guard stage [13]. The proposed mechanism for this impact was a reduced efficiency of chick provisioning at a crucial time in the breeding season, as more extensive sea ice increases the duration of the adults’ foraging trips and reduces the frequency at which the chicks are fed [13]. The negative impact of unusually extensive sea ice was also felt at the Pétrels Island colony at Pointe Géologie in the 2013/14 breeding season, with emaciated chicks often observed during the summer [19]. In this case, the negative impact of extensive sea ice was compounded by unusually warm air temperatures that caused snow melt and unprecedented amounts of rain [19]. Rain can be fatal to Adélie penguin chicks, as their downy plumage is not waterproof and when wet they may succumb to cold temperatures [19]. In the case of Pétrels Island, the compounded effects of extensive sea ice and warm temperatures were devastating, resulting in 100 % chick mortality in 2013/14 [19]. In the opposite scenario, at Pointe Géologie the size of breeding populations increased six years after a period of low sea ice extent and concentration [20]. As Adélie penguins commence breeding between 5 and 6 years of age, it is likely that sea ice conditions during the fledgling and yearling stages are important, with lower sea ice extent being favourable for young birds [20]. At Béchervaise Island adult penguins were also sensitive to extremes in sea ice concentrations in their winter foraging grounds, with either too much sea ice (>80 % cover) or too little sea ice (<15 % cover) negatively impacting adult survival [14]. Extreme climatic events that alter the sea ice environment may also impact Adélie penguin reproductive success. In 2010 there was a calving of the Mertz Glacier Tongue, which resulted in decreased polynya activity and sea ice production in the area [21, 22] and these local changes in the icescape appear to have negatively impacted Adélie penguin reproductive success at the nearby Pointe Géologie populations in the 2011/2012 and 2012/2013 breeding seasons [23].

While decadal scale monitoring studies have provided invaluable data on the short-term environmental sensitivities of Adélie penguin populations, given the long-term environmental change projected in the Southern Ocean, it is also prudent to consider the responses of populations to environmental change over longer time scales (e.g. thousands to tens of thousands of years) and during climate regime shifts, for example, during the transition from the last glacial maximum (LGM, 26–19.5 kya; [24]) to the Holocene warming period (11.7 kya–present) [25]. The responses of Adélie penguins to climate change during the LGM and Holocene have been well-studied in the Ross Sea and Antarctic Peninsula/Scotia Arc regions (Fig. 1) [26, 27]. In both locations, Adélie penguin numbers were much lower during the LGM than they are today, which was thought to be the result of reduced ice-free ground suitable for breeding [28, 29]. Phylogenetic studies have found evidence of two genetic lineages of Adélie penguins that are suggestive of two refuge populations dating to the LGM [29–31]. One of these lineages was comprised solely of individuals from modern Ross Sea colonies, suggesting that a refuge may have been situated somewhere in the vicinity of the Ross Sea during the LGM [31]. The second lineage was comprised of individuals from the Antarctic Peninsula, Scotia Arc, Ross Sea, and East Antarctica [29–31]. However, the samples analysed for the East Antarctic region were limited to two colonies at Gardner and Welch Islands and, given the length of the coastline, may not be representative of the genetic diversity of the broader region. As this second genetic lineage shows no strong geographic affinity, the location of its associated LGM refuge is unknown. Based on a genetic coalescent study, Adélie penguin numbers in the Antarctic Peninsula/Scotia Arc increased during the Holocene warming period from *ca*. 16 kya [29], roughly coincident with deglaciation of the region [32, 33]. In the Ross Sea, radiocarbon dated remains suggest that the Adélie penguin distribution expanded from approximately 8 kya, followed by two periods of reduced occupation from 5 to 4 kya and 2 to 1.1 kya [34].

The impacts of past climate regime shifts on Adélie penguins have been less well studied in East Antarctica. In the Windmill Islands and Vestfold Hills, radiocarbon dating of remains suggests that Adélie penguins were present by 9 kya and 8.5 kya respectively, roughly coincident with local deglaciation and, therefore, an emergence of available ice-free nesting habitat [35, 36]. There is also evidence for a peak in Adélie penguin numbers *ca*. 4 kya, coinciding with a mid-Holocene warm period [27, 36, 37]. During the LGM, the ancestors of individuals currently breeding at Gardner Island in the Prydz Bay region and Welch Island on the Mawson Coast were restricted to a refuge population along with the ancestors of extant Antarctic Peninsula/Scotia Arc and Ross Sea colonies [29–31]. However, as mentioned previously, the location of this refuge is unknown. Several questions regarding the past dynamics of East Antarctic Adélie penguin populations remain to be answered, including the trends in abundance during and after the LGM, whether an LGM refuge population may have been located in this area, and how populations may have responded to environmental changes across the extensive East Antarctic region. In this study we sought to address these questions using genetic data from multiple extant Adélie penguin colonies across East Antarctica.

## Results

### Summary statistics

We sequenced 56 individuals from six colonies located in East Antarctica (Béchervaise Island, Macey Island, Welch Island, Blakeney Point, Holl Island and Pétrels Island; Fig. 1). A 642 bp fragment of the mitochondrial hypervariable region (HVR) was sequenced for each individual [GenBank: KT932437 - KT932492], and a 902 bp fragment of cytochrome *b* (CytB) was sequenced for 45 individuals [GenBank: KT932493 - KT932537].

Genetic diversity was high for the HVR, with 85 polymorphic sites in the 642 bp fragment, 55 unique haplotypes recorded from the 56 individuals sequenced, and a mean number of pairwise differences between haplotypes of 8.54 ± 4.01. CytB genetic diversity was lower than for the HVR, with only 11 unique haplotypes among the 45 individuals sequenced. We did not detect any significant genetic structure among the colonies (CytB: F_ST_ = 0.023, *p* = 0.170; HVR: F_ST_ = 0.019, *p* = 0.055). The AMOVA analyses for HVR indicated that 98.07 % of the observed genetic variation occurred within colonies, with only 1.93 % of variation among colonies. The CytB result was similar, with 97.65 % of the observed genetic variation within colonies and 2.35 % of variation among colonies. These findings indicate that the East Antarctic samples can be analysed as a single population with respect to demographic history. The lack of genetic structure among East Antarctic colonies is consistent with a previous study of Adélie penguin genetic structure, which found genetic homogeneity among Adélie penguins around the continent based on seven microsatellite loci [38].

### East Antarctic Adélie penguin abundance over the past 22,000 years

**Fig. 2:**
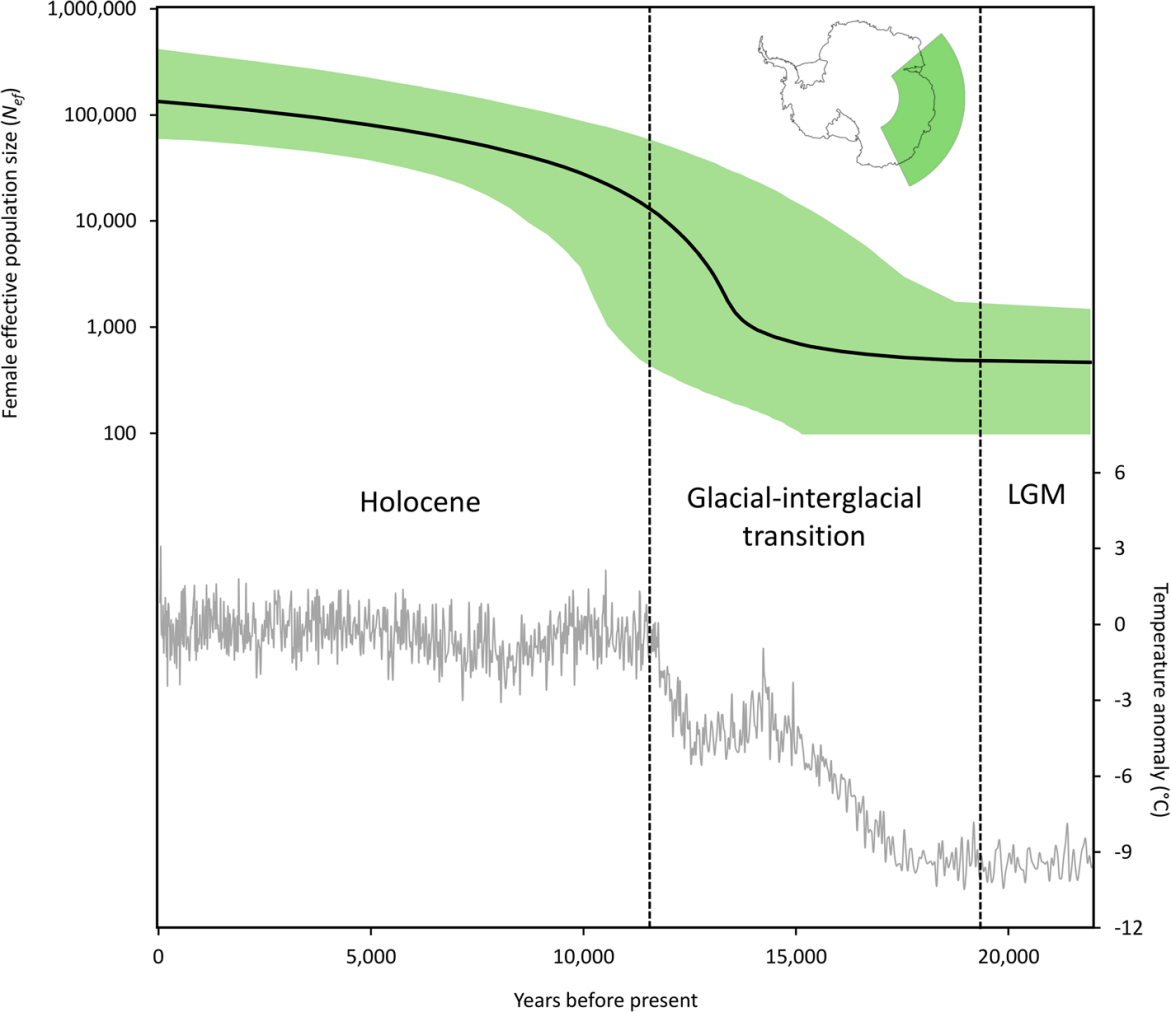
Abundance trend of East Antarctic Adélie penguins over the last 22,000 years. Extended Bayesian skyline plot showing the change in effective female population size (*N*_*ef*_), with the black line indicating the median estimate and the colour block showing the 95 % highest posterior density interval. The grey line indicates the East Antarctic temperature anomaly (the difference from the average of the last 1000 years) as estimated from the EPICA Dome C ice core [25]

There is evidence of a population expansion in East Antarctic Adélie penguins commencing around 14 kya, towards the end of the glacial-interglacial transition (Fig. 2). Interestingly, during the period of 22 kya to 15 kya, which encompassed the LGM and the majority of the subsequent glacial-interglacial transition period, the *N*_*ef*_ of East Antarctic Adélie penguins was less than 1000, and based on the 95 % highest posterior density interval (HPD) may have been zero (Fig. 2). It is therefore possible that Adélie penguins were not present in East Antarctica prior to *ca*. 15 kya. The population size then began to rapidly increase *ca*. 14 kya (95 % HPD: 11 kya – 19 kya), with a total increase of approximately 135-fold (Fig. 2). This period of population growth is coincident with increasing temperatures and deglaciation in East Antarctica during the glacial-interglacial transition and early Holocene (Fig. 2).

### Antarctic wide mtDNA phylogeny

**Fig. 3:**
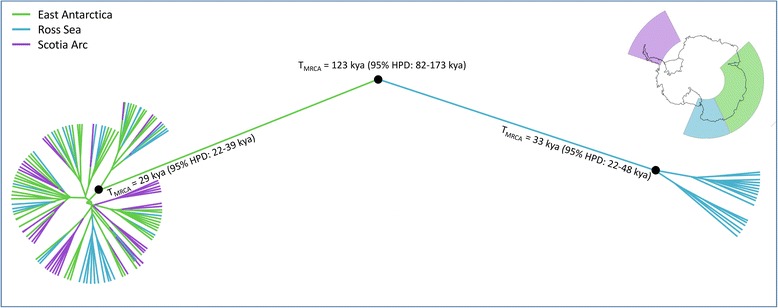
Phylogeny of Adélie penguins. East Antarctic penguins are indicated by green, Ross Sea penguins by blue, and Scotia Arc penguins by purple. Black dots indicate strongly supported clades, with a posterior probability of one. The time to most recent common ancestor for the strongly supported clades is indicated as T_MRCA_, with the 95 % highest posterior density interval shown in brackets

Our phylogenetic analysis of Adélie penguins from East Antarctica, the Ross Sea [31] and the Scotia Arc [29], revealed two strongly supported clades (posterior probability = 1; Fig. 3). One of these clades is comprised solely of penguins sampled at Ross Sea colonies, whereas the other contains individuals from colonies in East Antarctica, the Scotia Arc and the Ross Sea. This confirms the pattern found by Ritchie et al. (2004), who noted the presence of one Ross Sea lineage and one “Antarctic” lineage, which comprised individuals from the Ross Sea, Welch and Gardner Islands in East Antarctica, and Torgersen Island on the Antarctic Peninsula. Clucas et al. (2014) compared Scotia Arc penguins to Ross Sea individuals using a haplotype network, and found that individuals from the Scotia Arc also fell into the “Antarctic” lineage. Our study now shows that Adélie penguins from additional locations in East Antarctica, including Pétrels Island, Holl Island, Macey Island, Blakeney Point and Béchervaise Island, also fall into the “Antarctic” lineage.

Our dated phylogeny indicates that both lineages originated during the last glacial period and probably represent two glacial refugia, with the time to the most recent common ancestor (T_MRCA_) of the Ross Sea lineage estimated at 33 kya (95 % HPD: 22–48 kya), and of the Antarctic lineage estimated at 29 kya (95 % HPD: 22–39 kya).

Within the two lineages there is a high degree of topological uncertainty, suggesting very little genetic structure among Adélie penguin populations, aside from the division into two lineages. This is consistent with our F_ST_ and AMOVA results, as well the published findings of genetic homogeneity among Adélie penguins around much of the continent based on microsatellite DNA markers [38].

## Discussion

Mitochondrial DNA sequence data has revealed that Adélie penguin numbers in East Antarctica increased 135-fold during the most recent glacial-interglacial transition. This dramatic expansion suggests that the East Antarctic environment is currently much more favourable for Adélie penguins than it was prior to 14 kya. Our genetic analyses shows that the East Antarctic population began to expand between 19 and 11 kya (95 % HPD), with a median estimate of 14 kya. This was coincident with a global change in climate regime, from the LGM (26–19.5 kya) to the warm Holocene (11.7 kya–present) [25]. During this transitional period, glaciers and ice sheets retreated [39–41], Southern Ocean primary productivity increased by between two and five fold [42–44], the winter sea ice field is estimated to have halved in areal extent [45], and sea ice became seasonal, rather than perennial [46]. These changes in the East Antarctic coastal environment would have created more favourable conditions for Adélie penguins, by increasing the amount of ice-free ground suitable for nesting, by increasing prey abundance as marine productivity rose, and by creating more accessible marine foraging grounds and breeding habitat as sea ice concentrations lessened.

Previous studies based on radiocarbon dated remains, found at the Windmill Islands and Vestfold Hills, placed the occupation of East Antarctica by Adélie penguins at *ca*. 9 kya [35, 36]; our data from a more extensive set of sites across the region now show that East Antarctica was probably colonised at least 2000 years earlier. Given the confidence interval on our demographic reconstructions (Fig. 2), it is unclear whether Adélie penguins persisted in East Antarctica throughout the LGM in small numbers, or if they colonised the region during the post-glacial period from refugia located elsewhere.

Our phylogenetic analyses (Fig. 3) provide support to the notion that only two Adélie penguin refugia existed in Antarctica during the LGM [30, 31]. One refuge was most likely located near the Ross Sea [31], whereas the location of the other refuge remains unknown, but was clearly the source population for extant colonies in the Scotia Arc [29], parts of the Ross Sea [30, 31], and the full breadth of East Antarctica (Fig. 3). Given the high genetic differentiation of this lineage from the Ross Sea lineage, it seems unlikely that the second refuge was located in the vicinity of the Ross Sea, directing attention toward the Scotia Arc or East Antarctica as potential refuge locations.

Our results indicate a median expansion time of the East Antarctic population *ca*. 14 kya, whereas the Scotia Arc population is estimated to have expanded slightly earlier (*ca*. 17 kya, median estimate; [29]), coincident with the earlier deglaciation of that region compared to East Antarctica [32, 33, 41]. Given that the Scotia Arc population expanded first, it may be that the LGM refuge was located in this region and, as the lineage expanded and colonised new areas, it made its way to East Antarctica *ca*. 14 kya. The South Shetland Islands, in the Scotia Arc, had a similar glacial extent during the LGM as they do today [33], and could therefore have supported a small refuge population of Adélie penguins prior to widespread deglaciation of the Scotia Arc region from 18 kya. Under this scenario, Adélie penguins from a Scotia Arc refuge could have colonised an ice-free area in East Antarctica, such as the Vestfold Hills [47], Lützow-Holm Bay [48] or the Larsemann Hills [49] *ca*. 14 kya, subsequently becoming more widespread in East Antarctica as the rest of the region deglaciated from 12 kya [41]. Previous genetic analysis of radiocarbon dated remains indicate that both Adélie penguin lineages were present in the Ross Sea from at least *ca*. 6 kya, suggesting that individuals from the second refuge had spread to the Ross Sea by this time [30, 31].

It is also possible that an Adélie penguin refuge was located in East Antarctica. While most of the East Antarctic coastline was covered by glacial ice until at least 12 kya, some areas have been ice-free since before the LGM [41, 47] and could, theoretically, have acted as a refuge for small Adélie penguin breeding colonies if local sea ice and foraging conditions were favourable. If this were the case, then these sites were likely to be isolated pockets suitable for breeding Adélie penguin populations rather than broad regions of suitable breeding and marine foraging habitat. Previous studies have indicated that ice-free oases during the LGM were located at several sites within East Antarctica, including at the Vestfold Hills (based on lake sediment records; [47]), at Lützow-Holm Bay (based on Holocene raised beach deposits; [48]) and at the Larsemann Hills (based on radiocarbon dated moss deposits; [49]). The Bunger Hills were also partially ice-free during and since the LGM [50], however, the region is bounded by ice shelves and is therefore inaccessible to penguins. If Adélie penguins were present in East Antarctica at one of these sites before 14 kya, they may have expanded their range and numbers in the region as ice-free habitat became more plentiful coincident with the widespread retreat of the East Antarctic Ice Sheet from *ca*. 12 kya [41]. While there is evidence that these sites were ice-free and could have theoretically supported breeding Adélie penguins, the existence of Adélie penguin glacial refugia in East Antarctica or the Scotia Arc could be confirmed by radiocarbon dating of penguin remains from these potential refugia sites. The Vestfold Hills, Lützow-Holm Bay and the South Shetland Islands are all home to penguin breeding colonies today and, while no penguins currently breed at the Larsemann Hills, they may have done so in the past.

While ice-free ground suitable for nesting is a key requirement for the existence of Adélie penguin colonies, the species also requires accessible marine foraging grounds and sufficiently abundant prey to survive. Unlike winter during the LGM, when sea ice extent was double the current winter values, the LGM summer sea ice extent is estimated to have been similar to that seen in summer today [45]. Therefore, adult Adélie penguins provisioning for their chicks during the summer breeding season may have encountered similar sea ice extents as they do today. However, the penguins’ capacity to forage successfully would largely depend on the amount of fast ice present, which can impede their ability to reach foraging grounds [13, 19, 51]. The LGM sea ice records refer to total sea ice extent, with no differentiation between fast and pack ice [45], therefore it is unknown whether the LGM summer fast ice conditions would have been suitable for Adélie penguin foraging. Coastal polynyas could have facilitated Adélie penguin foraging amidst the sea ice field and also acted as hot spots of primary productivity [52]. Polynyas are known to have existed in several locations in the Weddell and Ross Seas during the LGM [52–56]. There are currently no records of LGM polynyas in East Antarctica, but this is more likely a result of the sparse sediment core record rather than an actual absence of polynyas, which are thought to have been more widespread during the LGM due to increased katabatic winds compared to today [53, 56].

Sea ice extent and seasonality began to shift from LGM conditions *ca*. 10.4 kya in Prydz Bay, with Holocene sea ice conditions similar to today’s reached between 10 and 9 kya [40]. This decline in sea ice occurred after the initial increase in abundance of East Antarctic Adélie penguins (*ca*. 14 kya; Fig. 2), suggesting that sea ice conditions were not the primary driver of population expansion. Recent studies in East Antarctica have shown that sea ice variation is a key driver of Adélie penguin population dynamics and key demographic parameters over yearly and decadal time scales [13, 18, 20, 57, 58], and sea ice declines are predicted to result in decreasing numbers of Adélie penguins in the most northerly latitudes of their breeding range over the coming decades [59]. It appears, however, that changes in sea ice extent and seasonality during the glacial-interglacial transition were not the key driver of East Antarctic Adélie penguin population expansion. This suggests that environmental drivers of population trends over thousands of years may differ to drivers over years or decades.

Increases in primary productivity did not commence until between 12 and 10 kya off MacRobertson Land [60, 61], and from *ca*. 10 kya off Adélie Land [42], indicating that changes in primary productivity were not an initial driver of the Adélie penguin abundance increase either. As Adélie penguins began increasing from 14 kya, this suggests that prey abundance was already sufficiently high for penguin survival prior to the increase in East Antarctic primary productivity between 12 and 10 kya. This is supported by evidence from another meso-predator, the Weddell seal, which persisted in East Antarctica throughout the LGM and post-glacial period in similar numbers to today [62]. For the Weddell seal’s population size in the region to be unchanged, the species must have had sufficiently abundant prey over this period; therefore, it is plausible that prey resources for the Adélie penguin, which breeds at the same time of year as the Weddell seal, were also sufficient prior to 14 kya. The overall indication, based on the timing of the East Antarctic Adélie penguin population increase, is that deglaciation, leading to increased ice-free area for nesting, was the initial environmental variable that changed sufficiently to allow the post-glacial abundance increase. Subsequent changes to sea ice and primary productivity after the initial population expansion may have sustained this trend in unison with increasing breeding habitat availability.

The observed post-glacial expansion of Adélie penguins is common to many penguin species, including emperors [63], kings [64], gentoos and chinstraps [29], which all had populations smaller in size and restricted in range during the LGM, and which expanded post-glacially. Interestingly, the increase in abundance of East Antarctic Adélie penguins began earlier and was far greater than that of the sympatric, closely related emperor penguins [62], which were also restricted to refugia during the LGM [63]. The East Antarctic emperor penguin population increased in abundance during the Holocene; however, the expansion was only 5.7 fold and did not commence until *ca.* 10 kya [62], approximately 4000 years after the Adélie penguin expansion. This suggests that the two species were influenced by different environmental drivers during the post-glacial period. While both species responded positively to declining ice, for the Adélie penguins it is likely that terrestrial ice sheet retreat was the key factor, whereas the expansion of emperor penguins was more closely coupled with reductions in sea ice extent [63].

## Conclusions

Our study has shown that East Antarctic Adélie penguins responded similarly to Scotia Arc Adélie penguins during climate warming following the LGM, with both populations undergoing an increase in population size coincident with an expansion of ice-free breeding habitat. Increases in Adélie penguin numbers in these two regions occurred asynchronously, in line with local deglaciation, indicating that Adélie penguins are sensitive to local glacier and ice sheet retreat. As climate change progresses, glaciers and ice sheets in Antarctica are expected to retreat further. This study highlights the possibility that, in regions where sea ice and prey conditions remain favourable, Adélie penguin numbers may expand in line with this deglaciation as additional breeding sites become exposed. In the Ross Sea, such an expansion has already occurred in response to receding glacial ice at the Beaufort Island colony, with an increase of 84 % in the Adélie penguin population between 1983 and 2010, concurrent with a 543 m retreat of the glacier field [65]. While the future trends in Adélie penguin abundance remain uncertain, our study suggests that ice sheet retreat and the availability of ice-free breeding areas may be overriding factors in determining the millennial scale abundance trends of this species. However, for Adélie penguin populations to expand in line with increasing breeding habitat, prey must be abundant and accessible enough to meet the requirements of the expanding population. Whether this will be the case in the future remains to be seen, as the impacts of climate change on Adélie penguin prey species, such as Antarctic krill (*Euphausia superba*), are currently ambiguous [8, 66, 67].

Our study also demonstrates that the environmental drivers underlying changes in population size over yearly, decadal and millennial timescales are not necessarily the same. In the case of East Antarctic Adélie penguins, sea ice conditions are a critical factor in population success over decadal and yearly timescales, however, deglaciation appears to have been the key driver of population change over millennia. This finding has important implications for the forecasting of species’ abundance and distribution changes under future climate change scenarios, and highlights the need to consider millennial scale trends alongside contemporary data for understanding species’ responses to climate change.

## Methods

### Field collections

Specimens of muscle tissue were collected between 2012 and 2014 from the carcasses of recently deceased Adélie penguins at Blakeney Point (*n* = 13), Holl Island (*n* = 7), Macey Island (*n* = 2), Welch Island (*n* = 13), Béchervaise Island (*n* = 11) and Pétrels Island (*n* = 10) (Fig. 1). We refer to these collections as the East Antarctic region samples. Tissue was transported and stored at −20 °C.

### Molecular laboratory

Genomic DNA was extracted using the QIAGEN DNeasy Blood and Tissue Kit following the manufacturer’s protocols. The mitochondrial hypervariable region (HVR) and cytochrome B (CytB) were sequenced, as these markers have been successfully used to reconstruct the demographic history of the closely related emperor penguin [63], and the use of HVR allowed for comparisons with published Adélie penguin datasets from the Ross Sea [30, 31] and Antarctic Peninsula/Scotia Arc [29].

HVR was amplified and sequenced for all individuals using primers AP1STR (5′-CCACCCTATACATACAATTCCCCTCCC-3′) [29] and H-A650 (5′-CTGACATAGGAACCAGAGGCGC-3′) [29, 31, 68]. CytB was amplified and sequenced for individuals from Béchervaise Island (n = 11), Macey Island (n = 1), Welch Island (n = 11), Blakeney Point (n = 12), and Pétrels Island (n = 10) using primers CytB-F1 (5′-ACTGCAGACACAACCCTAGC-3′) [63] and CytB-R1 (5′-GGGAAGAGGATCAGGAGGGT-3′) [63]. For both HVR and CytB, reaction mixes consisted of 7.5 μL of GoTaq Green Master Mix (Promega), 0.2 μM of each primer, and 5–10 ng of gDNA, made up to 15 μL with ddH_2_O. Annealing temperatures for HVR and CytB PCRs were 52.5 and 60 °C, respectively. Bi-directional Sanger sequencing using the PCR primer pairs was carried out at the Australian Genome Research Facility (AGRF). Occasional heteroplasmic sites were present in the HVR dataset, as expected for Adélie penguin HVR [29, 69], and these were re-scored according to IUPAC ambiguity codes when the secondary peak was >40 % of the height of the primary peak in both forward and reverse sequences.

### Summary statistics

jModeltest [70] was used to estimate the optimal nucleotide substitution model for each dataset and Arlequin v3.5 [71] was used to calculate summary statistics for the CytB and HVR datasets and to perform analyses of molecular variance (AMOVA).

### East Antarctic demographic reconstructions

The demographic history of East Antarctic Adélie penguins over the past 22,000 years was reconstructed using the coalescent extended Bayesian skyline plot method [72] within BEAST v2.1.3 [73]. The nucleotide substitution model for both HVR and CytB were specified as HKY [74] with four gamma categories, which was selected by jModelTest [70] as the optimal model in both cases. A strict molecular clock was used with lognormal substitution rate priors specified for HVR (mean = 0.55 substitutions/site/Myr, SD = 0.15) to reflect the published substitution rate [69]; and for CytB (mean = 0.039 substitutions/site/Myr, SD = 1.5) to reflect the substitution rate of CytB in the closely related emperor penguin [62].

The posterior distribution of effective population size through time was generated using the Markov chain Monte Carlo (MCMC) sampling procedure, which was run for 120 million generations with samples drawn every 5000 steps. Tracer v1.5 was used to visualise the sampling trace and to check the effective sample size values (ESS) to confirm convergence, with most ESS values exceeding 1000, and all values exceeding 200. Three independent BEAST analyses with different random number seeds were performed to ensure reproducibility of the posterior distribution.

The effective population size (*N*_*e*_) [75] is the number of individuals in an ideal population (i.e. a population with an equal sex ratio, random mating and no variation in reproductive success) that undergoes random genetic change at the same rate as the real population [76]. Because real populations do not usually adhere to these idealised constraints, *N*_*e*_ is almost always considerably smaller than the census size of a population (*N*); a recent review across a wide range of taxonomic groups found a median *N*_e_/*N* ratio of 0.14 [77]. Because mitochondrial DNA was used for this study, our estimate is for female effective population size (*N*_*ef*_). To convert the population size parameter of the demographic model (*N*_ef_*tau) to *N*_*ef*_, we divided the parameter by the generation length of Adélie penguins, which we have estimated at 13.3 years using the formula *tau* = A + [S/(1-S)], where A = age of first breeding (estimated at 4.85 for females, [78]) and S = yearly survival rate after reaching breeding age (estimated at 0.894, [51]). Generation length can be difficult to assess accurately for penguins, as both age at first breeding and annual survival rates may differ by location and be influenced by anomalous environmental conditions. It should therefore be noted that any variance in our estimate of *tau* would affect the absolute value of *N*_*ef*_ in our results, but has no bearing on either the timing or magnitude of the abundance increase reported. It should also be noted that the measure of *N*_*ef*_ applies to the total breeding population and is not necessarily constrained to our study area, which may be inside the boundaries of a larger panmictic breeding population.

### Antarctic wide phylogeny

In order to determine the phylogenetic placement of East Antarctic Adélie penguins within the global population we constructed a phylogeny for a dataset of HVR sequences of Adélie penguins from East Antarctica (this study), from the Scotia Arc and Antarctic Peninsula (hereinafter referred to as Scotia Arc) [29], and from the Ross and Somov Seas (hereinafter referred to as Ross Sea) [31]. The Scotia Arc dataset consisted of 36 individuals from the South Shetland Islands, the South Orkney Islands, the South Sandwich Islands, and Lagotellerie Island (Fig. 1) [GenBank PopSet: 634224762; [29]]. The Ross Sea dataset consisted of 49 individuals, randomly chosen from within GenBank PopSet: 45443792, which contains Adélie penguins sampled from Cape Adare, Port Martin, Adélie Cove, Edmonson Point and the Balleny Islands (Fig. 1) [31].

Bayesian phylogenetic analyses were performed on this dataset using BEAST v2.1.3 [73]. The nucleotide substitution model and substitution rate prior for HVR were specified as described above for the demographic reconstruction, and the coalescent Bayesian skyline tree prior was used [79]. The posterior distribution of phylogenetic trees was generated using the MCMC sampling procedure, which was run for 200 million iterations with trees logged every 40,000 steps. Tracer v.1.5 was used to check effective sample size (ESS) values to confirm convergence with all values >200. Four independent BEAST analyses, from different random number seeds, were performed to ensure reproducibility of the posterior distribution. The maximum clade credibility tree (after a burn in of 5 %) was selected using TreeAnnotator v2.1.3 and then visualised in FigTree v1.4. The heights specified for each node in the tree are median values.

## Availability of supporting data

The nucleic acid sequences supporting the results of this article are available in the GenBank repository, accession numbers: KT932437 - KT932537.
